# Systematic pan-cancer analysis identifies PIK3R3 as a lipid metabolism and immune regulation prognostic biomarker

**DOI:** 10.1515/med-2026-1439

**Published:** 2026-07-22

**Authors:** Zhixun Ding, Duandan Li

**Affiliations:** Department of Oncology, The Second Affiliated Hospital of Hunan University of Chinese Medicine, Changsha, Hunan, China; Department of Respiratory Medicine, Changsha Hospital of Traditional Chinese Medicine, Changsha, China

**Keywords:** pan-cancer, PIK3R3, gene set enrichment analysis, immune infiltration

## Abstract

**Objectives:**

We aimed to explore the critical role of PIK3R3 in pan-cancer, which to facilitate the development of more targeted prognostic markers and therapeutic strategies for these malignancies.

**Methods:**

In our study, pan-cancer transcriptome datasets were retrieved from Xena database. Subsequently, utilizing the Wilcoxon test, univariate Cox regression analysis as well as Kaplan-Meier (K-M) survival analysis to identify cancer types with high relevance to PIK3R3. Clinical metadata were stratified for correlation analysis, followed by differential expression gene (DEG) identification, overrepresentation analysis, and various functional analysis methods.

**Results:**

Lower-grade glioma (LGG), kidney renal clear cell carcinoma (KIRC), liver hepatocellular carcinoma (LIHC), lung adenocarcinoma (LUAD), and uterine corpus endometrial carcinoma (UCEC) exhibited strong associations with PIK3R3. PIK3R3 was closely linked to tumor grade stratification in LGG and UCEC and likely regulated these cancers via pathways such as lipid metabolism. Notably, UCEC showed high-level PIK3R3 mutations, mainly involving mutation and amplification. Compounds including Tetrachlorodibenzodioxin (TCDD), 1-Methyl-4-phenylpyridinium (MPP+), Estradiol, and Valproic Acid were predicted to target PIK3R3.

**Conclusions:**

Our results provide novel insights into the molecular mechanisms underlying PIK3R3-associated tumorigenesis and progression, which may contribute to the development of more targeted prognosis markers and therapeutic strategies for these malignancies.

## Introduction

Cancer stands as a leading cause of mortality and a major barrier to quality of life worldwide, with no definitive cure currently available [1]. In recent years, cancer immunotherapy has emerged as a prominent treatment modality, particularly immune checkpoint blockade therapy [2]. Even though substantial progress has been achieved in cancer treatment, including immunotherapy, target therapy, and radiotherapy, the 5-year overall survival (OS) of patients remains less than satisfactory [3]. With the continuous development and enrichment of public databases like Xena, pan-cancer expression analysis of genes, coupled with evaluation of their correlations with clinical prognosis and relevant signaling pathways, which has become a viable approach to uncover novel immunotherapeutic targets [4].

Phosphoinositide-3-kinase regulatory subunit 3 (PIK3R3), also known as p55PIK and a component of the PI3K regulatory domain. The PIK3R3 protein, encoded by the PIR3R3 gene, establishes a connection with the p110 catalytic subunit via the iSH2 domain. In contrast to other regulatory subunits, PIK3R3 is distinguished by a distinct NH2 terminal. Previous research uncovered that the NH2 terminal of PIK3R3 is responsible for mediating functions specific to PIK3R3, which set it apart from other regulatory subunits. This mediation occurs through its binding to crucial cell – growth proteins such as the retinoblastoma protein (RB1) and the proliferating cell nuclear antigen (PCNA) [5]. PIK3R3 was demonstrated upregulated in various cancers and plays critical roles in tumorigenesis, cell proliferation, and metastasis. For instance, in liver cancer, PIK3R3 is upregulated and activates Akt signaling to control cancer growth by regulating CDKN1C and SMC1A. In colorectal cancer, PIK3R3 induces epithelial-to-mesenchymal transition (EMT) and promotes metastasis. Additionally, PIK3R3 is regulated by LACTB to promote autophagy and inhibit EMT and proliferation through the PI3K/AKT/mTOR signaling pathway [5], 6]. Notably, as the activation of PIK3R3 has been shown to decrease hepatosteatosis, it has emerged as a promising novel target for developing therapies against non-alcoholic fatty liver disease (NAFLD) and metabolic syndrome [7]. Collectively, growing evidence highlights PIK3R3 as a key gene in tumor biology, with its expression significantly impacting patient prognosis.

Nevertheless, most existing studies on the role of PIK3R3 in tumors have been confined to single cancer types, with no pan-cancer investigations of its associations with diverse malignancies. Here, we leveraged the Xena database to analyze PIK3R3 expression levels and their prognostic significance across multiple cancer types. We further explored potential correlations between PIK3R3 expression and clinical traits, molecular regulatory mechanisms, pathway enrichment, and immune infiltration levels of 22 immune cell types. Additionally, mutational landscape analyses and drug regulation network construction were performed to dissect the biological functions of PIK3R3 in tumors. This study aims to provide comprehensive insights into the role of PIK3R3 across various cancers.

## Methods

### Data collection

In this research, an integrated pan-cancer transcriptome sequencing dataset was obtained from the Xena database, which is based on the comprehensive genomic datasets from The Cancer Genome Atlas (TCGA), Therapeutically Applicable Research To Generate Effective Treatments (TARGET), and Genotype-Tissue Expression (GTEx) projects [8]. The dataset was employed to identify tumor types with a high degree of relevance where the PIK3R3 gene exerts a significant impact on both the etiology and prognosis of the disease. Upon the identification of the highly relevant tumor types, we specifically retrieved the complete cohort information for each cancer type from the TCGA and TARGET databases. After the exclusion of normal control samples and those lacking in survival status or survival time samples, we further refined the phenotypic information associated with each dataset, which was then utilized for further in-depth analysis.

### Identification of cancer types with high relevance to PIK3R3

In the quest to delineate the spectrum of cancers that manifest a profound association with PIK3R3 at the pathogenic level, we harnessed a comprehensive pan-cancer dataset and embarked on an initial horizontal assessment of PIK3R3’s expression profiles across a diverse array of tumors. Subsequently, utilizing the Wilcoxon test, we evaluated the expression levels of PIK3R3 for statistically significant differences between the tumor group and the control group within each tumor type. Another aspect of the assessment focused on the prognosis of tumors. The optimal cut-off value was determined within a rational range using the ‘survival’ package, following which all samples were stratified into high and low expression groups based on this value; Kaplan-Meier (K-M) survival analysis was subsequently performed for further prognostic assessment [9]. This approach was undertaken with the intent to discern the intimate association of PIK3R3 expression with the prognostic outcomes in various types of cancer. Subsequently, a multivariate Cox regression model was further constructed on the basis of the positive features screened out by univariate Cox regression analysis with a statistical significance of p<0.05, and an independent prognostic model was ultimately established, which could quantify the independent prognostic effects of each factor on the clinical outcomes of patients with the corresponding cancer type.

### Clinical relevance analysis of PIK3R3

Following the identification of highly relevant cancer types, to further explore the clinical relevance of PIK3R3, we subsequently collected information on various levels for each type of tumor: 1) patient age (grouped by the demarcation of 65 years old); 2) patient race; 3) patient gender; 4) patient BMI (categorized according to World Health Organization standards [10]); 5) tumor progression and prognostic assessment indicators (including pathologic T, pathologic M, pathologic N, grade, and stage); 6) history of radiotherapy (yes or no). After stratifying the information as described above, we employed the Wilcoxon test method to determine in which groups there were significant differences in gene expression levels of PIK3R3, thereby assessing its clinical relevance.

### Exploration of the common regulatory mechanisms of PIK3R3 in highly relevant cancer types

To explore the molecular regulatory mechanisms commonly mediated by PIK3R3 across all highly relevant cancer types, we utilized the featureCount data from the transcriptome sequencing of these cancer cohorts. Samples were stratified into high- and low-PIK3R3 expression groups based on the median expression level of PIK3R3. Subsequently, we conducted differential expression analysis between these groups using the ‘DESeq2’ package to identify differentially expressed genes (DEGs) [11]. The thresholds for differential analysis were set with an absolute fold change greater than 1.5 and a p-value less than 0.05. Then, we sought the intersection among the DEGs that were upregulated and downregulated, excluding PIK3R3 itself from this process, thereby identifying genes that are significantly regulated by PIK3R3 across all highly relevant cancer types. Relying on the identified genes, we further conducted overrepresentation analysis (ORA) based on the Gene Ontology (GO) and Kyoto Encyclopedia of Genes and Genomes (KEGG) databases. Additionally, we performed a correlation analysis between these genes and PIK3R3.

### Enrichment analysis of PIK3R3 in highly relevant cancer types

To investigate the pathways and molecular mechanisms in which PIK3R3 is involved from a holistic tumor perspective, this project leveraged the original results of the differential analyses for each highly relevant cancer type. Genes were ranked based on their log2FoldChange values, followed by Gene Set Enrichment Analysis (GSEA) based on the ‘clusterProfiler’ package to identify the regulatory mechanisms that are highly associated with PIK3R3 in that specific cancer type [12]. The analysis thresholds were set with an absolute value of the Normalized Enrichment Score (NES) greater than 1, p-value less than 0.05, and q-value less than 0.25.

### Immune-related analyses of PIK3R3 in highly relevant cancer types

We explored the specific pathways and influence of PIK3R3 in the immune context of various highly relevant cancer types from both micro and macro perspectives. At the micro level, we employed the CIBERSORT algorithm to conduct an immune infiltration analysis of 22 immune cell types within the tumor microenvironment, examining the abundance differences between the high-PIK3R3 expression group and the low-PIK3R3 expression group, stratified by the median expression level of PIK3R3 [13]. Concurrently, we performed a correlation analysis between PIK3R3 and the abundance of each immune cell type using the Pearson method. Subsequently, we utilized the ESTIMATE and TIDE algorithms to compute the ESTIMATE Score, IMMUNE Score, STROMAL score, and TIDE Score, providing a macroscopic view of the tumor immune microenvironment. These scores served as a metric to evaluate the significance of PIK3R3 in relation to immune cell activity, stromal cell composition, and the mechanisms of immune evasion. Additionally, we utilized the Wilcoxon test and Pearson correlation analysis to screen for immune checkpoints that are highly correlated with PIK3R3, with the aim of providing a reference for research on the role of PIK3R3 in immunotherapy.

### Mutational landscape analysis of PIK3R3 in highly relevant cancer types

Gene mutations are one of the significant factors in the occurrence and progression of cancer. This study conducted an investigation of the mutational status of PIK3R3 in highly relevant cancer types using the cBioportal [14]. Initially, we observed the mutation and copy number variation of PIK3R3 across various tumors. Subsequently, we obtained the Microsatellite Instability (MSI) scores and Tumor Mutational Burden (TMB) scores for each tumor sample and performed a Pearson correlation analysis.

### Construction of the PIK3R3 interaction network and drug regulation network

In the culmination of our research, we employed geneMANIA to assess the mechanisms of PIK3R3 at the interaction level from perspectives such as Co-expression, Physical Interaction, Genetic interaction, Shared protein domains, Co-localization, and Pathway, and constructed a regulatory network [15]. Furthermore, leveraging the Comparative Toxicogenomics Database (CTD), we predicted drugs that could target PIK3R3 and delineated a drug regulation network [16].

### Statistical analysis

This study was conducted using R version 4.4.0 for analysis. Correlation analysis was performed using the Pearson method, and significant intergroup differences were compared using the Wilcoxon method. Correlation scatter plots, volcano plots, and other graphical representations were created using ggplot2, while heatmaps were generated with the ComplexHeatmap package, and network diagrams were constructed using the visNetwork package [17], 18].

## Results

### Five types of cancer with a high correlation to PIK3R3 were identified

**Figure 1:**
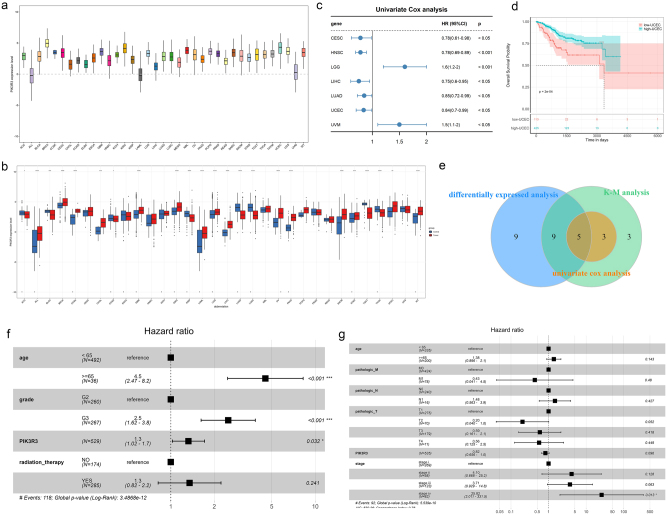
Identification of cancer types with high correlation to PIK3R3 (a) expression level of PIK3R3 in pan-cancer. (b) Differential expression analysis. (c–d) Univariate Cox regression analysis and Kaplan-Meier (K-M) survival analysis. (e) Venn diagram. Optimal multivariate Cox regression model for lower grade glioma (LGG, f) and kidney renal clear cell carcinoma (KIRC, g). *p<0.05; **p<0.01; ***p<0.001; ****p<0.0001; ns p>0.05.

To identify tumor types in which PIK3R3 is highly involved in the entire process of pathogenesis, progression, and deterioration, we conducted multidimensional analysis and evaluation of 32 types of cancer from both the perspective of significant gene expression and the correlation with tumor prognosis (Figure 1a). We found that, in terms of PIK3R3 expression levels, there were significant differences in expression across 23 types of cancer (cancer group vs. control group), and that PIK3R3 exhibited the highest relative expression in breast cancer (BRCA) (Figure 1b). In the evaluation at the prognostic level using univariate Cox regression analysis and K-M survival analysis, we identified 7 and 20 types of cancer, respectively (Figure 1c and d). Integrating the results of these analyses, we ultimately constructed a Venn diagram and identified 5 cancer types with high relevance, which are: Lower-Grade Glioma (LGG), Kidney Renal Clear Cell Carcinoma (KIRC), Liver Hepatocellular Carcinoma (LIHC), Lung Adenocarcinoma (LUAD), and Uterine Corpus Endometrial Carcinoma (UCEC) (Figure 1e). Further independent prognostic analyses revealed that PIK3R3, together with age, grade and radiation therapy, was integrated to construct the optimal multivariate Cox regression model in LGG and exhibited a significant independent prognostic value (p<0.05, Figure 1f) for this cancer type. In KIRC, PIK3R3 was also incorporated into the optimal multivariate Cox regression model along with age, stage, pathologic_M, pathologic_N and pathologic_T; although its independent prognostic effect did not reach the conventional statistical significance threshold of 0.05 (p=0.096, Figure 1g), it presented a marginally significant potential prognostic trend in this malignancy. Notably, the results of univariate Cox regression analysis for PIK3R3 showed no statistical significance in LIHC, LUAD and UCEC, a phenomenon that we speculate to be associated with the reduction in valid sample size caused by the exclusion of samples with incomplete clinical information during the performance of independent prognostic analyses.

### PIK3R3 may mediate the tumor progression process in KIRC and LUAD

**Figure 2:**
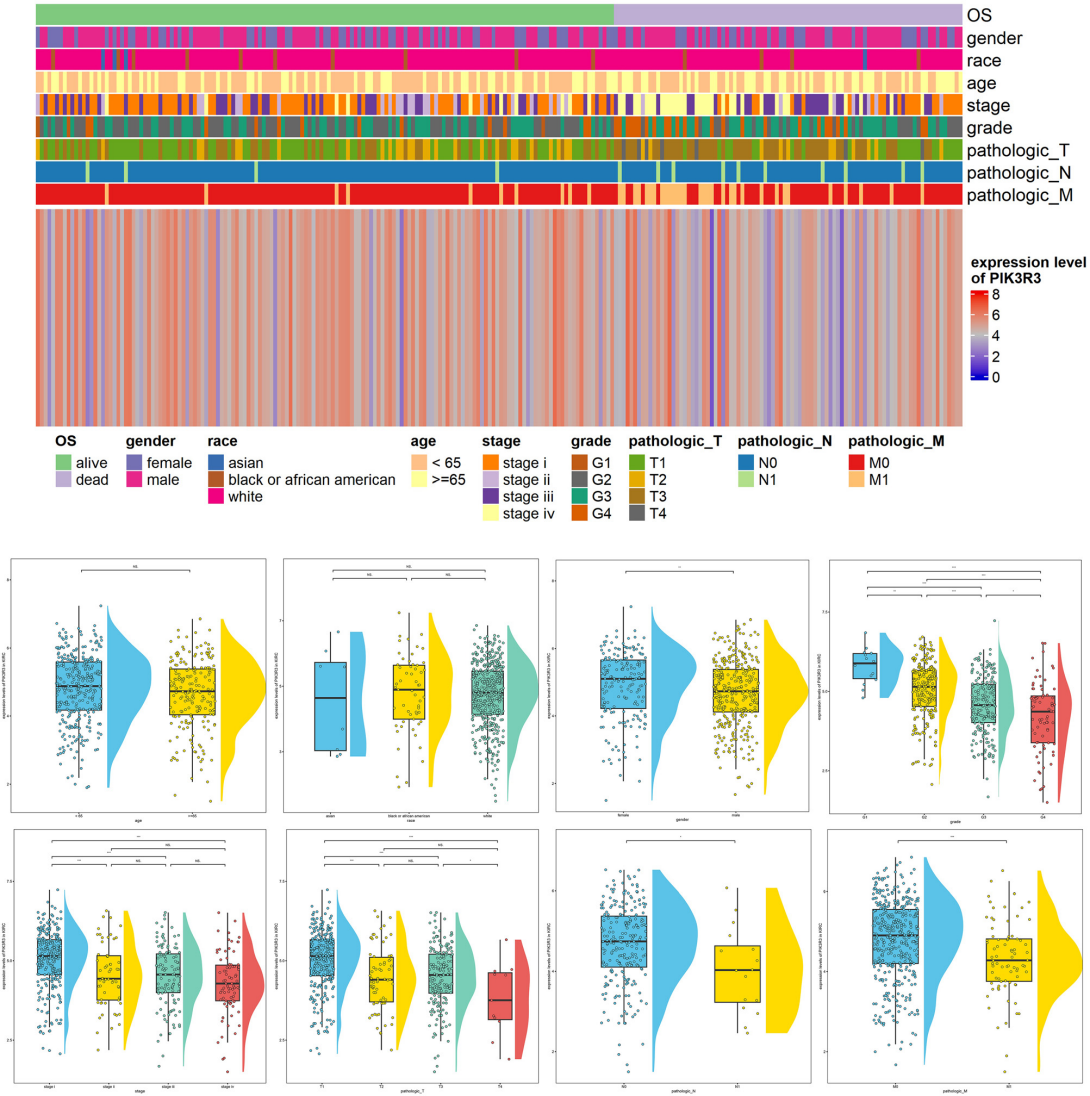
Clinical relevance analysis between PIK3R3 and clinical trait indicators.

A plethora of clinical trait indicators are currently utilized to delineate the characteristics of tumors and cancer patients at various levels and stages. Through clinical correlation analysis based on this clinical trait information, we have observed that PIK3R3 exhibits higher expression levels in female patients with KIRC and LUAD (Figure 2). Concurrently, it demonstrates a significant correlation with clinical indicators associated with tumor progression in these two types of malignancies. Furthermore, PIK3R3 exhibits a high degree of correlation with the grade stratification of tumors in LGG and UCEC. The results also indicate that radiotherapy significantly upregulates the expression levels of PIK3R3 in LGG, suggesting its potential involvement in a series of regulatory mechanisms induced by radiation therapy.

### PIK3R3 may regulate highly relevant cancer types by affecting lipid metabolism and other pathways

**Figure 3:**
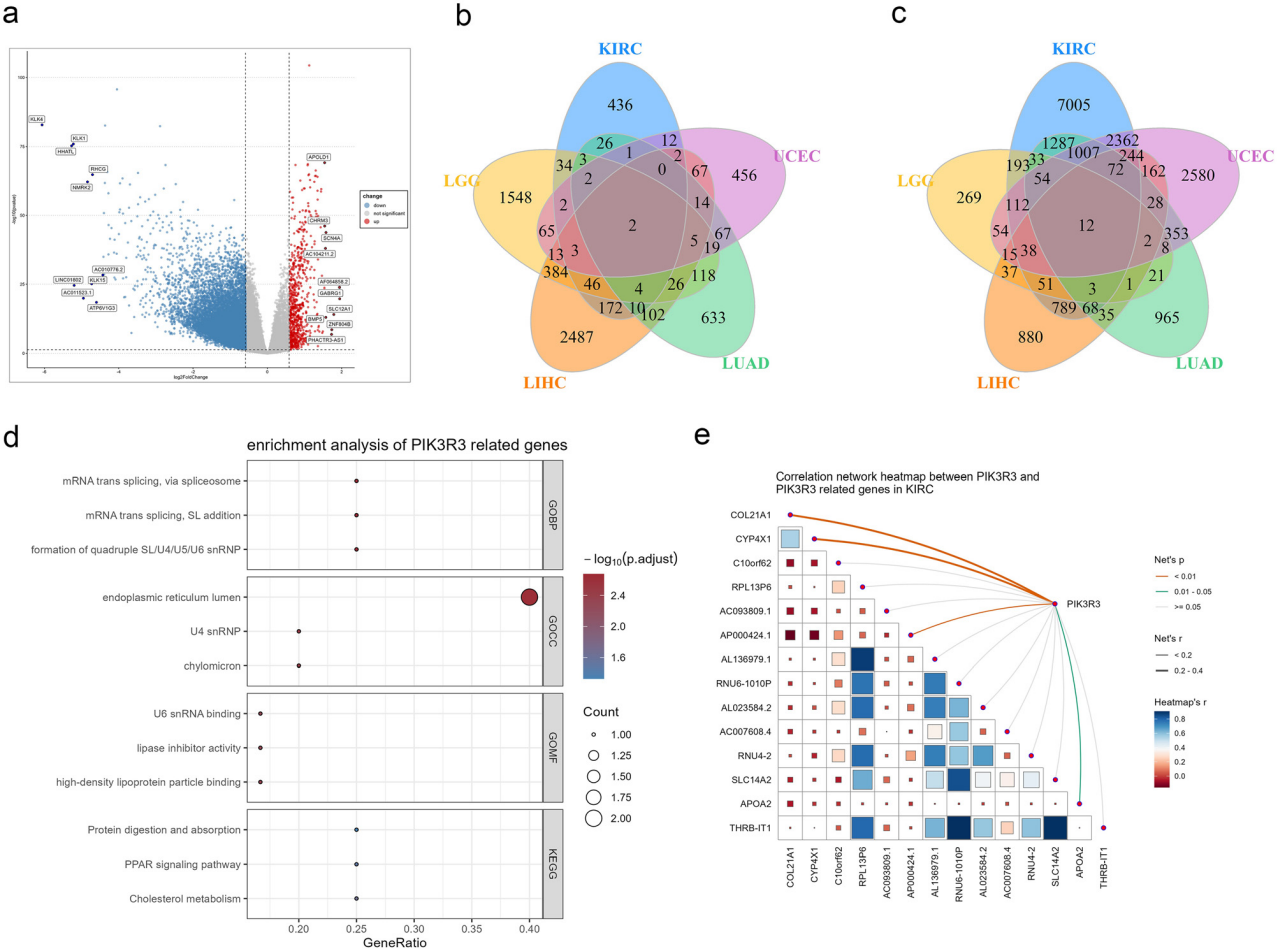
Enrichment analysis of PIK3R3 in pan-cancer (a) differential expression analysis. (b–c) Volcano plot and heatmap of DEGs. (d) Overrepresentation analysis. (e) Correlation analysis.

To further investigate whether PIK3R3 influences the occurrence and progression of five highly correlated cancer types through common regulatory mechanisms, we conducted differential expression analysis based on the transcriptome data of these five cancer types (Figure 3a). Then, we searched for common genes among the significantly upregulated DEGs and the significantly downregulated DEGs (Figure 3b and c). We have identified a total of 14 genes that are consistently regulated by PIK3R3 across the five highly correlated cancer types. The genes are as follows: COL21A1, CYP4X1, C10orf62, RPL13P6, AC093809.1, AP000424.1, AL136979.1, RNU6-1010P, AL023584.2, AC007608.4, RNU4-2, SLC14A2, APOA2, and THRB-IT1. In the subsequent enrichment analysis, we discovered that these genes are predominantly associated with the endoplasmic reticulum, lipid metabolism, and the PPAR signaling pathway (Figure 3d). Additionally, in the correlation analysis, we also found that PIK3R3 exhibits a high degree of correlation with COL21A1, C10orf62, and AC093809.1, all of which are significantly associated in the majority of cancer types with high correlation (Figure 3e).

**Figure 4:**
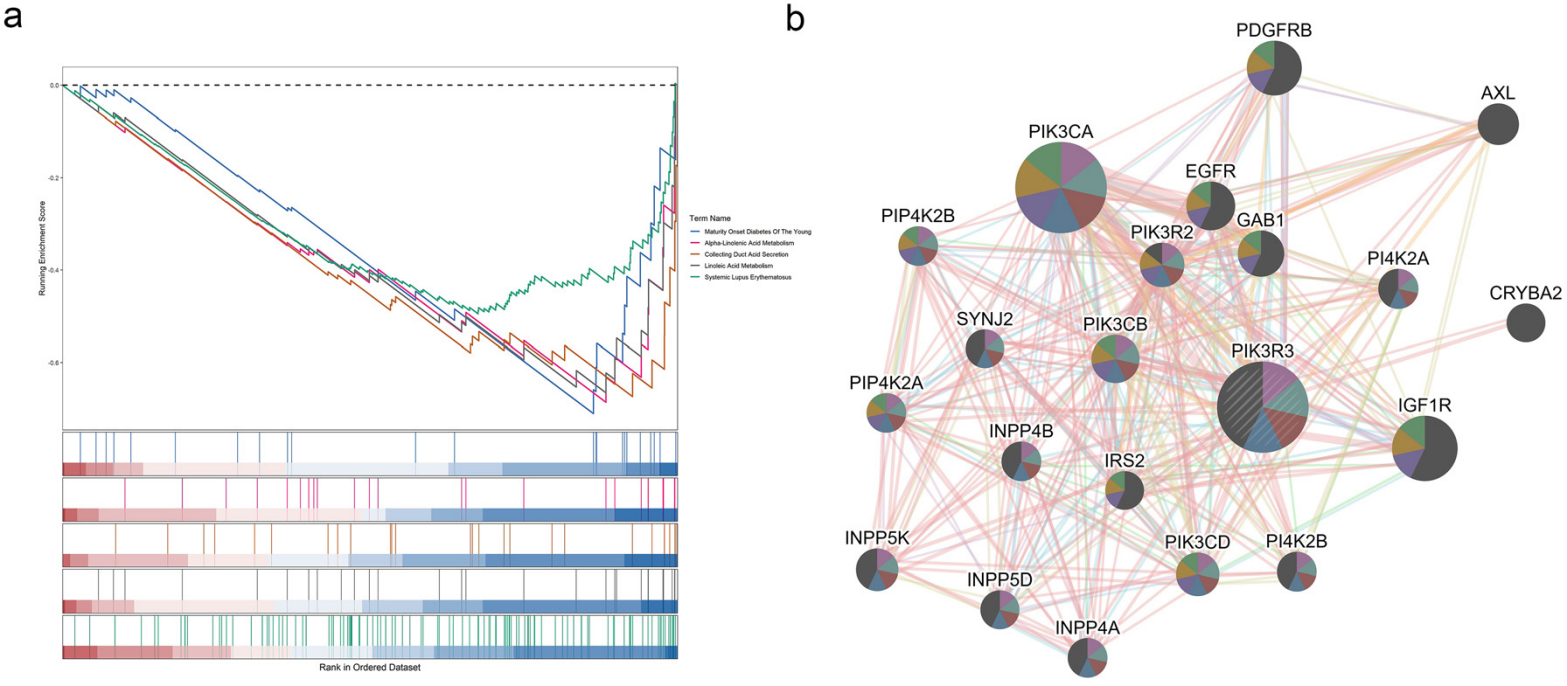
Single-gene GSEA enrichment analysis (a) and geneMANIA interaction network (b).

Similar to the results of the ORA enrichment analysis, in the single-gene GSEA enrichment analysis based on the KEGG database of the transcriptome sequencing data of various highly correlated cancer types, we also found a high correlation between PIK3R3 and lipid metabolism (Figure 4a). For instance, in KIRC, PIK3R3 is associated with the metabolism of alpha-linolenic acid and arachidonic acid, and in UCEC, it is related to fatty acid metabolism and degradation. In a striking parallel, our analysis of the geneMANIA interaction network of PIK3R3 also identified numerous entries related to lipid metabolism, such as “phospholipid biosynthetic process”, “glycerolipid biosynthetic process”, “lipid modification”, and “lipid phosphorylation” (Figure 4b).

### PIK3R3 regulates highly relevant cancer types through multiple immune pathways

**Figure 5:**
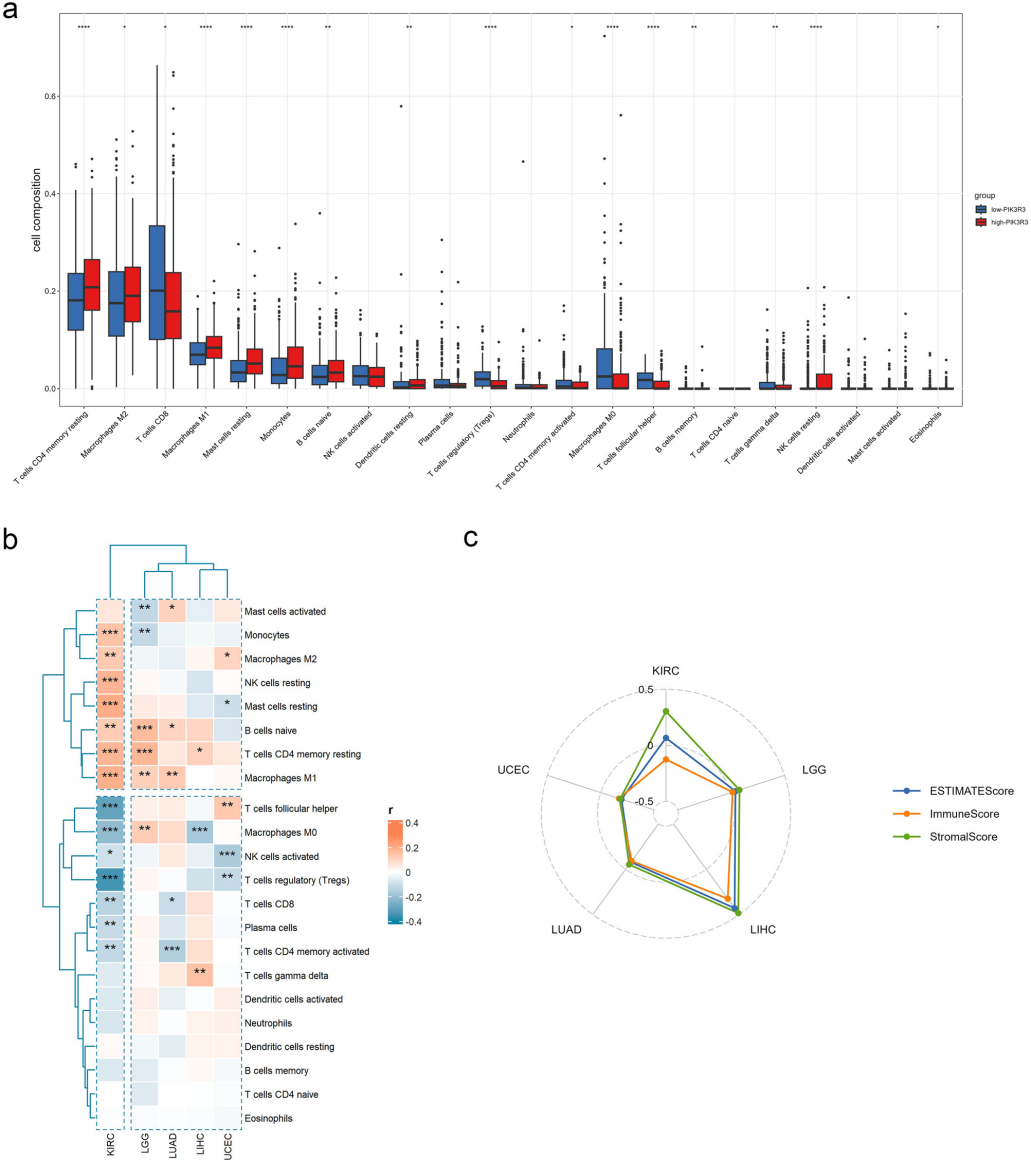
Immune infiltration analysis (a) comparison of the infiltration levels of 22 immune cell types. (b) Boxplot showing 16 immune cell types with significantly different infiltration levels. (c) Correlation analysis between PIK3R3 and immune cells. *p<0.05; **p<0.01; ***p<0.001; ****p<0.0001; ns p>0.05.

**Figure 6:**
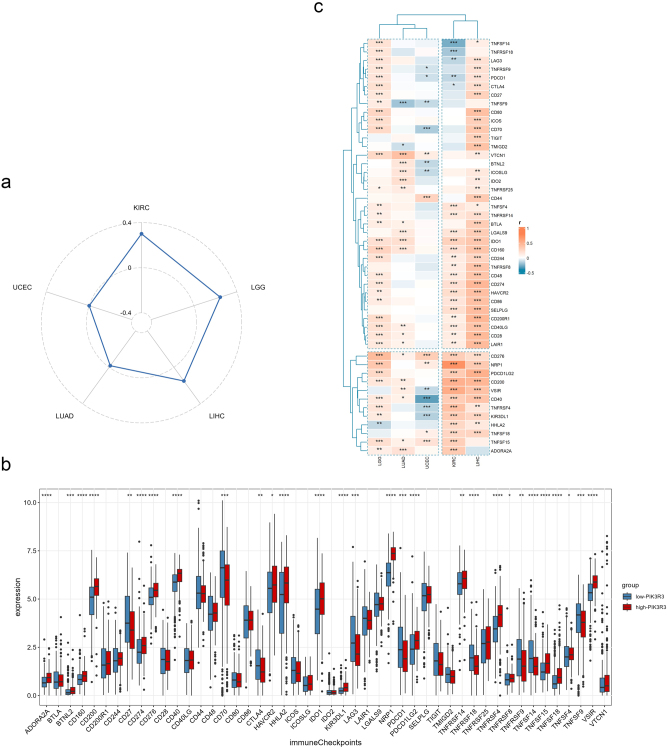
Correlation analysis between PIK3R3 and immune evasion response (a). Heatmap and immune checkpoints boxplot (b–c). *p<0.05; **p<0.01; ***p<0.001; ****p<0.0001; ns p>0.05.

Through immune infiltration analysis, we observed that PIK3R3 significantly influenced a variety of immune cells within the immune microenvironment of highly relevant cancer types, with the most extensive impact observed in KIRC, where the abundance of 16 types of immune infiltrating cells was highly correlated with PIK3R3. In our assessment of 22 types of immune infiltrating cells, immune cell populations such as T cells CD4 memory resting, B cells naive, and Macrophages M1 were found to be influenced by PIK3R3 across various types of tumors (Figure 5a and b). Furthermore, we identified a significant correlation between PIK3R3 and the stromal cells in KIRC and LIHC (Figure 5c). Additionally, there is a substantial correlation between PIK3R3 and the immune evasion response in KIRC and LGG (Figure 6a). Ultimately, we also examined the immune checkpoints influenced by PIK3R3 in each of the highly relevant cancer types and discovered that a substantial number of immune checkpoints were affected by PIK3R3 across the five highly correlated cancer types, with LIHC exhibiting the most extensive influence, involving up to 43 immune checkpoints (Figure 6b and c).

### PIK3R3 exhibits high-level mutations in UCEC

**Figure 7:**
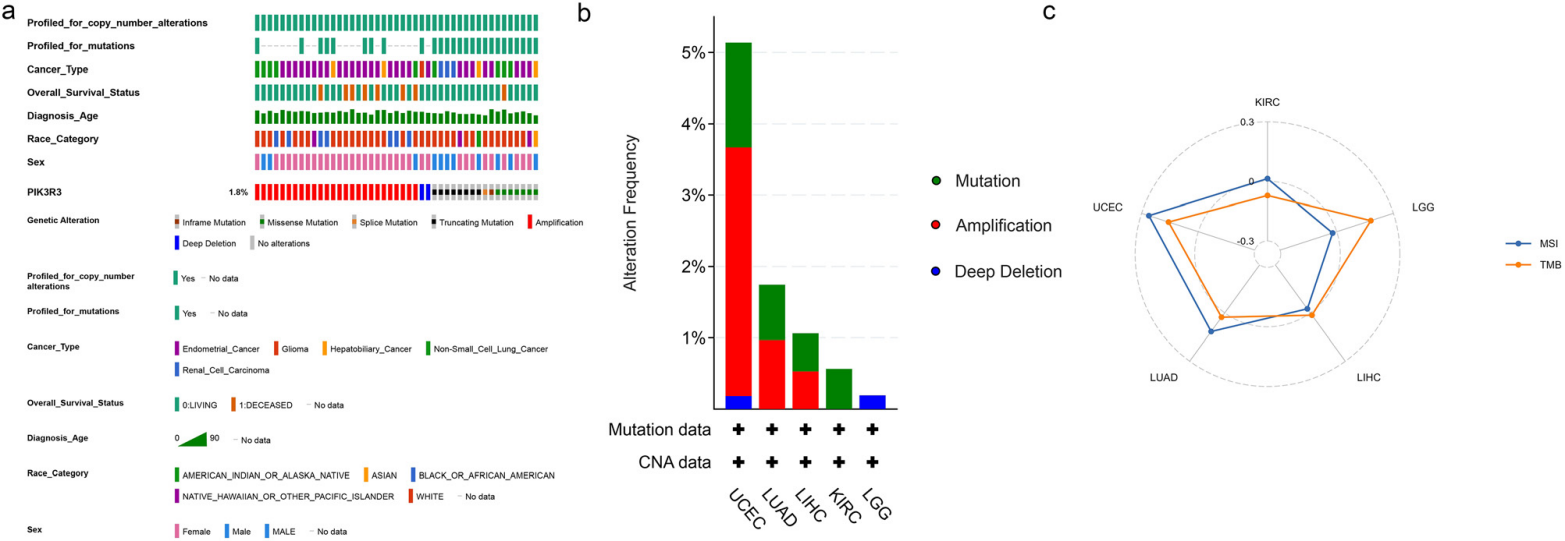
Analysis of tumor microenvironment (a–b) genetic mutations. (c) Microsatellite instability with PIK3R3.

Genetic mutations are a significant pathway in tumor pathogenesis. Through our analysis, we have identified varying degrees of mutations in the PIK3R3 gene across UCEC, LUAD, LIHC, and KIRC, with the highest frequency observed in UCEC (Figure 7a and b). Concurrently, we have observed that the mutational spectra of PIK3R3 predominantly involve Mutation and Amplification, with a minor subset experiencing Deep Deletion. Furthermore, we have discovered that there is a certain association between PIK3R3 and microsatellite instability in UCEC (Figure 7c).

### Potential drug prediction for targeting PIK3R3

**Figure 8:**
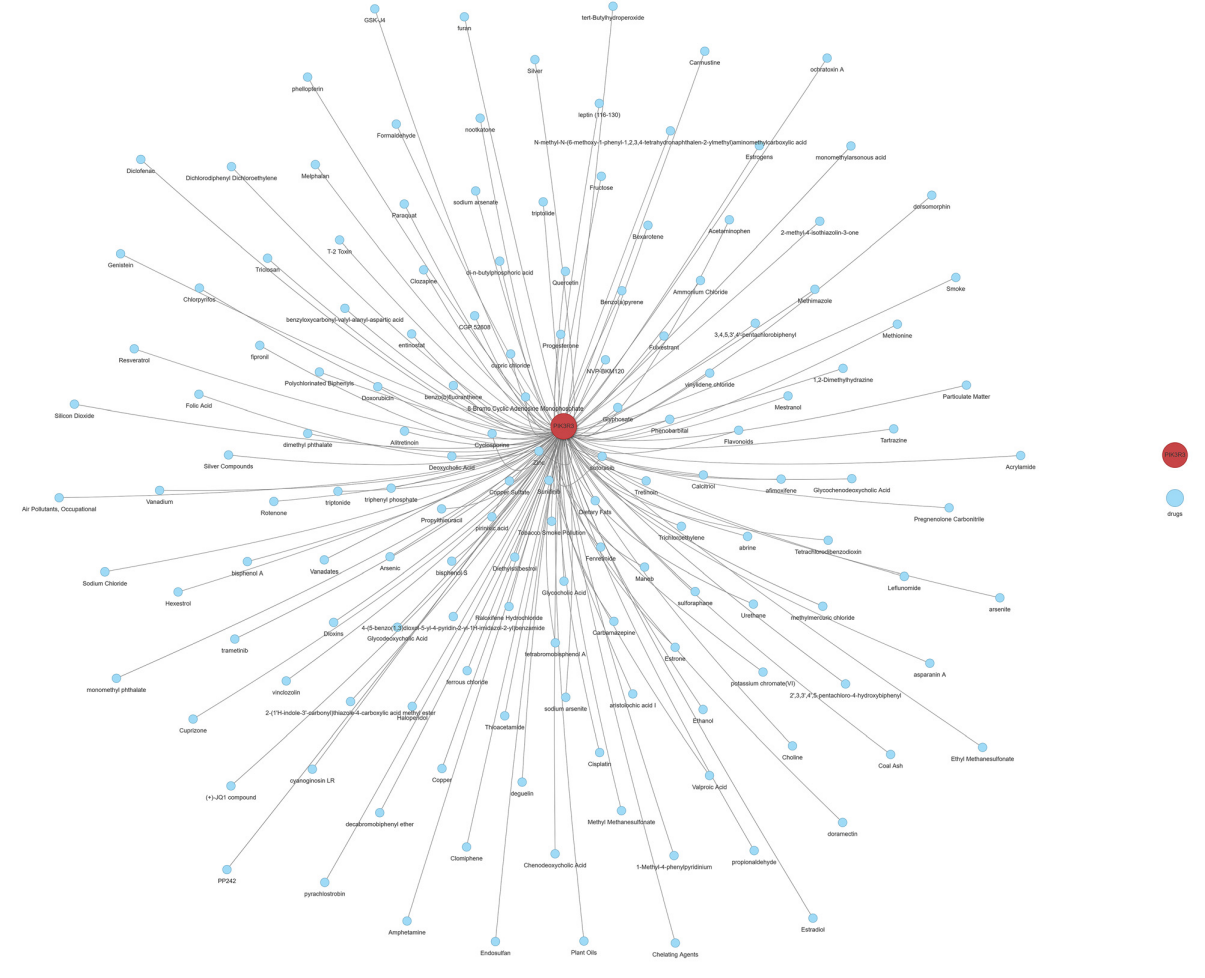
Potential drug prediction for targeting PIK3R3.

Ultimately, leveraging the drug prediction outcomes from the CTD database, we have identified compounds with high potential for research that are hypothesized to be associated with PIK3R3. Among these compounds, those supported by empirical evidence include: Tetrachlorodibenzodioxin (TCDD), 1-Methyl-4-phenylpyridinium (MPP+), Estradiol, Valproic Acid, bisphenol A, and Paraquat (Figure 8).

## Discussion

In the present study, we found the PIK3R3 gene is highly expressed in 15 types of cancer, with the highest expression in the BRCA group, as so as PIK3R3 was highly correlated with LGG, KIRC, and LIHC, among others. The results for gastric cancer, ovarian cancer, and liver cancer were similar to those of previous research [19], [20], [21]. As the second most common cancer worldwide, BRCA underscores the critical importance of early diagnosis and prognostic management. Our findings reveal that gene PIK3R3 exhibits the highest expression in BRCA, aligning with a 2025 study by Cai et al. on BRCA. Their results demonstrated that patients with high-expressed PIK3R1 protein had better outcomes in terms of DFS and OS, when breast cancer was at an early stage (stage I/II) [22]. Collectively, our results supplement the limited understanding of PIK3R3 in BRCA and hold promise for developing PIK3R3-targeted strategies to enhance prognostic prediction and therapeutic precision for cancer patients.

Additionally, it was found that PIK3R3 expression was associated with gender in specific cancer types. Higher PIK3R3 expression was observed in female patients with KIRC and LUAD, which was significantly correlated with clinical indicators of tumor progression, suggesting potential implications for guiding immunotherapy selection across gender groups [4]. A strong correlation between PIK3R3 expression and tumor grade stratification in LGG and UCEC was further revealed. Previous reports have indicated that PIK3R3 expression in BRCA cells is linked to pathological stage progression, collectively highlighting PIK3R3 as a potential biomarker for prognostic assessment across multiple cancer types [23]. Furthermore, our study is the first to investigate the tripartite relationship among radiation therapy, PIK3R3, and cancer. We identified a significant association between PIK3R3 expression and radiation therapy, suggesting that PIK3R3 levels may serve as a potential biomarker for predicting radiation therapy response in cancer patients.

Furthermore, prior work has demonstrated that metabolic and immune-related genes can serve as prognostic biomarkers across cancers, including glioma metabolic regulation [24], immune regulatory biomarkers identified through integrative transcriptomic and single-cell approaches [25], 26], and pro-tumor prognostic genes acting via inflammatory pathways [27]. The further enrichment analysis revealed a high correlation between PIK3R3 and the lipid metabolism pathway. For instance, processes such as phospholipid biosynthetic process”, “glycerolipid biosynthetic process”, “lipid modification”, and “lipid phosphorylation” were all found to be related to PIK3R3. The latest research has emphasized that the phospholipid biosynthetic pathway plays a crucial role in the comprehensive regulation of nucleotide metabolism, redox balance, and the properties of cellular defense membranes [28]. Dória’s research demonstrates that phospholipid biosynthetic pathways are dynamically regulated during mammary epithelial cell differentiation and exhibit a significant correlation with breast cancer patient survival outcomes [29]. Furthermore, glycerolipid are regarded as new types of cellular signal transduction entities, playing a crucial role as signal transmission nodes in many physiological and pathological processes [30]. The glycerolipid metabolism had been regarded as potential diagnostic biomarkers for acute pancreatitis [31]. Membrane lipid modification displayed biological and therapeutic potential in tumors [32]. Early studies on high-fat diet-induced fatty liver phenotypes in mice have demonstrated that PIK3R3 overexpression promotes hepatic fatty acid oxidation via PIK3R3-induced PPARα expression. Hepatic PIK3R3 knockout in normal mice leads to increased liver triglyceride levels, highlighting the critical role of the PIK3R3-HNF4α-PPARα signaling axis in hepatic lipid metabolism [7]. In liver cancer cells, PIK3R3 regulates lipid metabolism and hepatoma cell proliferation by modulating PPARA expression [33]. Our data are consistent with previously published findings, further underscoring the significant association between PIK3R3 and lipid metabolism while providing novel insights for cancer therapy.

More importantly, our results not only reinforce this metabolic link but also extend to reveal that PIK3R3 plays an essential role in cancer immunity. Our results showed that PIK3R3 plays an essential role in cancer immunity. Tumor-infiltrating immune cells have important impacts on the occurrence and development of tumors and can antagonize or promote tumor occurrence and development [34]. Tumor microenvironment (TME) features serve as markers for evaluating tumor cell responses to immunotherapy and influence clinical outcomes [35]. Specifically, PIK3R3 exerts a regulatory effect on the activation status of CD4 memory resting T cells and the polarization direction of M1 macrophages: by modulating the functional phenotype of these two core immune cell populations, PIK3R3 can reshape the immunosuppressive or immunostimulatory landscape of the TME, thereby affecting the proliferation, invasion and metastatic potential of tumor cells [36], 37]. Pearson correlation analysis revealed positive correlations between PIK3R3 and stromal cells in KIRC and LIHC. As a major component of the TME, stromal cells play critical roles in tumor metabolism, growth, metastasis, immune escape, and treatment resistance. These cells can be recruited from adjacent non-cancerous host stromal cells or formed via transdifferentiation from stromal cells or tumor cells. Additionally, they participate in tumorigenesis, progression, and drug resistance by secreting various factors and exosomes, regulate angiogenesis and tumor metabolism, and modulate immune responses within the TME and extracellular matrix [38]. In addition, bibliometric evidence highlights the rapidly expanding focus on immune checkpoint-related mechanisms and complications in oncology [39]. We observed a significant correlation between PIK3R3 expression and TIDE Score in KIRC and LGG, which implies that PIK3R3 may strengthen the immune escape capability of tumor cells through regulating the expression and signaling of key immune checkpoint molecules [40]. This mechanism further promotes the immune evasion of tumor cells and facilitates tumor progression. Our research further clarifies the broader tumor applicability of PIK3R3 and confirms that PIK3R3 expression is intricately involved in immune checkpoint biology across most cancer types, with particularly pronounced associations observed in LIHC. Chemotherapy was initially believed to have only immunosuppressive effects. However, an increasing number of studies have shown that its combined application with immune checkpoint inhibitors in the treatment of solid tumors can significantly enhance the anti-tumor response, highlighting the core hub role of immune checkpoints in the regulation of the cancer immune microenvironment [41]. Collectively, these findings indicate that PIK3R3 expression is closely linked to immune cell infiltration in tumors, influences patient prognosis, and highlights new targets for developing immunotherapeutic strategies.

In the era of precision medicine, TMB has emerged as a promising pan-cancer predictive biomarker for guiding immunotherapy [42]. Its utility in enhancing responses to immune checkpoint inhibitors has been validated in non-small cell lung cancer (NSCLC) and colorectal cancer (CRC), while also demonstrating prognostic value across multiple malignancies [43], 44]. In NSCLC, high nonsynonymous TMB (>8 mutations/Mb) was associated with favorable prognostic outcomes, including overall survival, disease-free survival, and lung cancer-specific survival. Additionally, TMB-high is associated with better prognosis in patients with colorectal cancer treated with drug chemotherapy. Concurrently, microsatellite instability as key biomarker in immune checkpoint inhibitor therapy, which has been established as an independent predictor of clinical outcomes in CRC [45]. Our study revealed that the PIK3R3 gene exhibits the highest mutation frequency in UCEC, primarily manifesting as mutations and amplifications, with discernible correlations between PIK3R3 and MSI status. The results showed that gene alterations of PIK3R3 may regulate the growth and progression of a variety of tumors, including UCEC [46]. Building upon existing literature and our novel observations, these findings suggest that PIK3R3 expression may modulate both TMB and MSI, thereby influencing patient responses to immunotherapy.

Furthermore, we preliminarily identified several compounds that are hypothesized to be associated with PIK3R3 using the CTD database, including Tetrachlorodibenzodioxin (TCDD), 1-Methyl-4-phenylpyridinium (MPP+), Estradiol, and Valproic Acid. These drug-gene associations provided potential starting points for subsequent experimental validation of PIK3R3-targeted therapies. For instance, Valproic Acid, a known histone deacetylase inhibitor [47], has been reported to exert antitumor effects in multiple cancer types [48], 49], and its potential regulatory relationship with PIK3R3 may provide a novel combinatorial therapeutic strategy for cancers with high PIK3R3 expression.

In summary, our first pan-cancer analysis of PIK3R3 reveals its differential expression between tumor and normal tissues, and its correlations with clinical features and radiotherapy responses. PIK3R3 acts as an independent prognostic factor in multiple malignancies, and may regulate tumor progression by modulating lipid metabolism. Moreover, PIK3R3 expression is associated with immune cell infiltration, immune checkpoints, TMB and MSI across cancers, with varying impacts on tumor immunity. Together, these findings deepen our understanding of PIK3R3 in tumorigenesis and provide insights for developing precise immunotherapeutic strategies.

Although our approach offers valuable mechanistic insights into pan-cancer pathogenesis and diagnostic strategies, the results must be interpreted with caution due to inherent limitations. While PIK3R3’s influence on immune infiltration and its associations with MSI and TMB suggest it is a promising target for immunotherapy, current findings require translation into clinical trial outcomes. Our CTD database-based prediction identified potential PIK3R3-associated compounds that require further experimental validation, including *in vitro* drug-target binding assays, cell viability assays in PIK3R3-high and PIK3R3-low cancer cell lines, and *in vivo* models to evaluate the therapeutic efficacy of candidate compounds. Further prospective clinical validation is essential to establish the robustness and clinical reliability of these findings across diverse cancer populations.

## What is known?

Most studies on PIK3R3 in tumors have focused on single cancer types, lacking pan-cancer investigations into its associations with diverse malignancies.

## What new information does this article contribute?

This study conducts the first comprehensive pan-cancer analysis of PIK3R3, identifying its prognostic relevance in 5 cancer types; it reveals PIK3R3’s links to clinical stratification and lipid metabolism, and predicts 4 targeted compounds, filling gaps in systematic pan-cancer research on PIK3R3.
